# Supplementary material for: The effects of genital myiasis on the diversity of the vaginal microbiota in female Bactrian camels
Source: BMC Vet Res. 2022 Mar 5;18:87. doi: 10.1186/s12917-022-03189-5 (PMC8897907; doi:10.1186/s12917-022-03189-5)
Supplement: Supplementary file 5 — Additional file 5. [file 12917_2022_3189_MOESM5_ESM.zip › MPL201709200_16s_yy/Treat1/css/js_css/jsPDF-master.zip!/jsPDF-master/docs/global.html]

Global - Documentation


## Home

### Classes

- jsPDF

### Global

- addFont
- addHTML
- addMetadata
- addPage
- autoPrint
- CapJoinStyles
- circle
- ellipse
- getFontList
- lines
- lstext
- output
- rect
- roundedRect
- save
- setDisplayMode
- setDrawColor
- setFillColor
- setFont
- setFontSize
- setFontStyle
- setLineCap
- setLineJoin
- setLineWidth
- setPage
- setProperties
- setTextColor
- text
- triangle

# Global

### Members

#### addHTML

Source:
:   - plugins/addhtml.js, line 12

Deprecated:
:   - This is being replace with a vector-supporting API. See
      this link

Renders an HTML element to canvas object which added to the PDF

This feature requires html2canvas
or rasterizeHTML

#### autoPrint

Source:
:   - plugins/autoprint.js, line 8

Makes the PDF automatically print. This works in Chrome, Firefox, Acrobat
Reader.

##### Example

```
var doc = new jsPDF()
doc.text(10, 10, 'This is a test')
doc.autoPrint()
doc.save('autoprint.pdf')
```

#### CapJoinStyles

Source:
:   - jspdf.js, line 2082

Is an Object providing a mapping from human-readable to
integer flag values designating the varieties of line cap
and join styles.

### Methods

#### addFont(Postscript, Name, Font) → {fontKey}

Source:
:   - jspdf.js, line 1883

Add a custom font.

##### Parameters:

| Name | Type | Description |
| --- | --- | --- |
| `Postscript` | String | name of the Font. Example: "Menlo-Regular" |
| `Name` | String | of font-family from @font-face definition. Example: "Menlo Regular" |
| `Font` | String | style. Example: "normal" |

##### Returns:

the (same as the internal method)

Type
:   fontKey

#### addMetadata(metadata, namespaceuri) → {jsPDF}

Source:
:   - plugins/xmp\_metadata.js, line 28

Adds XMP formatted metadata to PDF

##### Parameters:

| Name | Type | Description |
| --- | --- | --- |
| `metadata` | String | The actual metadata to be added. The metadata shall be stored as XMP simple value. Note that if the metadata string contains XML markup characters "<", ">" or "&", those characters should be written using XML entities. |
| `namespaceuri` | String | Sets the namespace URI for the metadata. Last character should be slash or hash. |

##### Returns:

Type
:   jsPDF

#### addPage() → {jsPDF}

Source:
:   - jspdf.js, line 1158

Adds (and transfers the focus to) new page to the PDF document.

##### Returns:

Type
:   jsPDF

#### circle(x, y, r, style) → {jsPDF}

Source:
:   - jspdf.js, line 1770

Adds an circle to PDF

##### Parameters:

| Name | Type | Description |
| --- | --- | --- |
| `x` | Number | Coordinate (in units declared at inception of PDF document) against left edge of the page |
| `y` | Number | Coordinate (in units declared at inception of PDF document) against upper edge of the page |
| `r` | Number | Radius (in units declared at inception of PDF document) |
| `style` | String | A string specifying the painting style or null. Valid styles include: 'S' [default] - stroke, 'F' - fill, and 'DF' (or 'FD') - fill then stroke. A null value postpones setting the style so that a shape may be composed using multiple method calls. The last drawing method call used to define the shape should not have a null style argument. |

##### Returns:

Type
:   jsPDF

#### ellipse(x, y, rx, rx, style) → {jsPDF}

Source:
:   - jspdf.js, line 1706

Adds an ellipse to PDF

##### Parameters:

| Name | Type | Description |
| --- | --- | --- |
| `x` | Number | Coordinate (in units declared at inception of PDF document) against left edge of the page |
| `y` | Number | Coordinate (in units declared at inception of PDF document) against upper edge of the page |
| `rx` | Number | Radius along x axis (in units declared at inception of PDF document) |
| `rx` | Number | Radius along y axis (in units declared at inception of PDF document) |
| `style` | String | A string specifying the painting style or null. Valid styles include: 'S' [default] - stroke, 'F' - fill, and 'DF' (or 'FD') - fill then stroke. A null value postpones setting the style so that a shape may be composed using multiple method calls. The last drawing method call used to define the shape should not have a null style argument. |

##### Returns:

Type
:   jsPDF

#### getFontList() → {Object}

Source:
:   - jspdf.js, line 1854

Returns an object - a tree of fontName to fontStyle relationships available to
active PDF document.

##### Returns:

Like {'times':['normal', 'italic', ... ], 'arial':['normal', 'bold', ... ], ... }

Type
:   Object

#### lines(lines, x, y, scale, style, closed) → {jsPDF}

Source:
:   - jspdf.js, line 1527

Adds series of curves (straight lines or cubic bezier curves) to canvas, starting at `x`, `y` coordinates.
All data points in `lines` are relative to last line origin.
`x`, `y` become x1,y1 for first line / curve in the set.
For lines you only need to specify [x2, y2] - (ending point) vector against x1, y1 starting point.
For bezier curves you need to specify [x2,y2,x3,y3,x4,y4] - vectors to control points 1, 2, ending point. All vectors are against the start of the curve - x1,y1.

##### Example

```
.lines([[2,2],[-2,2],[1,1,2,2,3,3],[2,1]], 212,110, 10) // line, line, bezier curve, line
```

##### Parameters:

| Name | Type | Description |
| --- | --- | --- |
| `lines` | Array | Array of *vector* shifts as pairs (lines) or sextets (cubic bezier curves). |
| `x` | Number | Coordinate (in units declared at inception of PDF document) against left edge of the page |
| `y` | Number | Coordinate (in units declared at inception of PDF document) against upper edge of the page |
| `scale` | Number | (Defaults to [1.0,1.0]) x,y Scaling factor for all vectors. Elements can be any floating number Sub-one makes drawing smaller. Over-one grows the drawing. Negative flips the direction. |
| `style` | String | A string specifying the painting style or null. Valid styles include: 'S' [default] - stroke, 'F' - fill, and 'DF' (or 'FD') - fill then stroke. A null value postpones setting the style so that a shape may be composed using multiple method calls. The last drawing method call used to define the shape should not have a null style argument. |
| `closed` | Boolean | If true, the path is closed with a straight line from the end of the last curve to the starting point. |

##### Returns:

Type
:   jsPDF

#### lstext(text, x, y, spacing) → {jsPDF}

Source:
:   - jspdf.js, line 1475

Deprecated:
:   - We'll be removing this function. It doesn't take character width into account.

Letter spacing method to print text with gaps

##### Parameters:

| Name | Type | Description |
| --- | --- | --- |
| `text` | String \| Array | String to be added to the page. |
| `x` | Number | Coordinate (in units declared at inception of PDF document) against left edge of the page |
| `y` | Number | Coordinate (in units declared at inception of PDF document) against upper edge of the page |
| `spacing` | Number | Spacing (in units declared at inception) |

##### Returns:

Type
:   jsPDF

#### output(type, options) → {jsPDF}

Source:
:   - jspdf.js, line 981

Generates the PDF document.

If `type` argument is undefined, output is raw body of resulting PDF returned as a string.

##### Parameters:

| Name | Type | Description |
| --- | --- | --- |
| `type` | String | A string identifying one of the possible output types. |
| `options` | Object | An object providing some additional signalling to PDF generator. |

##### Returns:

Type
:   jsPDF

#### rect(x, y, w, h, style) → {jsPDF}

Source:
:   - jspdf.js, line 1611

Adds a rectangle to PDF

##### Parameters:

| Name | Type | Description |
| --- | --- | --- |
| `x` | Number | Coordinate (in units declared at inception of PDF document) against left edge of the page |
| `y` | Number | Coordinate (in units declared at inception of PDF document) against upper edge of the page |
| `w` | Number | Width (in units declared at inception of PDF document) |
| `h` | Number | Height (in units declared at inception of PDF document) |
| `style` | String | A string specifying the painting style or null. Valid styles include: 'S' [default] - stroke, 'F' - fill, and 'DF' (or 'FD') - fill then stroke. A null value postpones setting the style so that a shape may be composed using multiple method calls. The last drawing method call used to define the shape should not have a null style argument. |

##### Returns:

Type
:   jsPDF

#### roundedRect(x, y, w, h, rx, rx, style) → {jsPDF}

Source:
:   - jspdf.js, line 1671

Adds a rectangle with rounded corners to PDF

##### Parameters:

| Name | Type | Description |
| --- | --- | --- |
| `x` | Number | Coordinate (in units declared at inception of PDF document) against left edge of the page |
| `y` | Number | Coordinate (in units declared at inception of PDF document) against upper edge of the page |
| `w` | Number | Width (in units declared at inception of PDF document) |
| `h` | Number | Height (in units declared at inception of PDF document) |
| `rx` | Number | Radius along x axis (in units declared at inception of PDF document) |
| `rx` | Number | Radius along y axis (in units declared at inception of PDF document) |
| `style` | String | A string specifying the painting style or null. Valid styles include: 'S' [default] - stroke, 'F' - fill, and 'DF' (or 'FD') - fill then stroke. A null value postpones setting the style so that a shape may be composed using multiple method calls. The last drawing method call used to define the shape should not have a null style argument. |

##### Returns:

Type
:   jsPDF

#### save(filename) → {jsPDF}

Source:
:   - jspdf.js, line 2156

Saves as PDF document. An alias of jsPDF.output('save', 'filename.pdf')

##### Parameters:

| Name | Type | Description |
| --- | --- | --- |
| `filename` | String | The filename including extension. |

##### Returns:

Type
:   jsPDF

#### setDisplayMode(zoom, layout, pmode) → {jsPDF}

Source:
:   - jspdf.js, line 1231

Set the display mode options of the page like zoom and layout.

##### Parameters:

| Name | Type | Description |
| --- | --- | --- |
| `zoom` | integer \| String | You can pass an integer or percentage as a string. 2 will scale the document up 2x, '200%' will scale up by the same amount. You can also set it to 'fullwidth', 'fullheight', 'fullpage', or 'original'.  Only certain PDF readers support this, such as Adobe Acrobat |
| `layout` | String | Layout mode can be: 'continuous' - this is the default continuous scroll. 'single' - the single page mode only shows one page at a time. 'twoleft' - two column left mode, first page starts on the left, and 'tworight' - pages are laid out in two columns, with the first page on the right. This would be used for books. |
| `pmode` | String | 'UseOutlines' - it shows the outline of the document on the left. 'UseThumbs' - shows thumbnails along the left. 'FullScreen' - prompts the user to enter fullscreen mode. |

##### Returns:

Type
:   jsPDF

#### setDrawColor(ch1, ch2, ch3, ch4) → {jsPDF}

Source:
:   - jspdf.js, line 1912

Sets the stroke color for upcoming elements.

Depending on the number of arguments given, Gray, RGB, or CMYK
color space is implied.

When only ch1 is given, "Gray" color space is implied and it
must be a value in the range from 0.00 (solid black) to to 1.00 (white)
if values are communicated as String types, or in range from 0 (black)
to 255 (white) if communicated as Number type.
The RGB-like 0-255 range is provided for backward compatibility.

When only ch1,ch2,ch3 are given, "RGB" color space is implied and each
value must be in the range from 0.00 (minimum intensity) to to 1.00
(max intensity) if values are communicated as String types, or
from 0 (min intensity) to to 255 (max intensity) if values are communicated
as Number types.
The RGB-like 0-255 range is provided for backward compatibility.

When ch1,ch2,ch3,ch4 are given, "CMYK" color space is implied and each
value must be a in the range from 0.00 (0% concentration) to to
1.00 (100% concentration)

Because JavaScript treats fixed point numbers badly (rounds to
floating point nearest to binary representation) it is highly advised to
communicate the fractional numbers as String types, not JavaScript Number type.

##### Parameters:

| Name | Type | Description |
| --- | --- | --- |
| `ch1` | Number \| String | Color channel value |
| `ch2` | Number \| String | Color channel value |
| `ch3` | Number \| String | Color channel value |
| `ch4` | Number \| String | Color channel value |

##### Returns:

Type
:   jsPDF

#### setFillColor(ch1, ch2, ch3, ch4) → {jsPDF}

Source:
:   - jspdf.js, line 1979

Sets the fill color for upcoming elements.

Depending on the number of arguments given, Gray, RGB, or CMYK
color space is implied.

When only ch1 is given, "Gray" color space is implied and it
must be a value in the range from 0.00 (solid black) to to 1.00 (white)
if values are communicated as String types, or in range from 0 (black)
to 255 (white) if communicated as Number type.
The RGB-like 0-255 range is provided for backward compatibility.

When only ch1,ch2,ch3 are given, "RGB" color space is implied and each
value must be in the range from 0.00 (minimum intensity) to to 1.00
(max intensity) if values are communicated as String types, or
from 0 (min intensity) to to 255 (max intensity) if values are communicated
as Number types.
The RGB-like 0-255 range is provided for backward compatibility.

When ch1,ch2,ch3,ch4 are given, "CMYK" color space is implied and each
value must be a in the range from 0.00 (0% concentration) to to
1.00 (100% concentration)

Because JavaScript treats fixed point numbers badly (rounds to
floating point nearest to binary representation) it is highly advised to
communicate the fractional numbers as String types, not JavaScript Number type.

##### Parameters:

| Name | Type | Description |
| --- | --- | --- |
| `ch1` | Number \| String | Color channel value |
| `ch2` | Number \| String | Color channel value |
| `ch3` | Number \| String | Color channel value |
| `ch4` | Number \| String | Color channel value |

##### Returns:

Type
:   jsPDF

#### setFont(fontName, fontStyle) → {jsPDF}

Source:
:   - jspdf.js, line 1820

Sets text font face, variant for upcoming text elements.
See output of jsPDF.getFontList() for possible font names, styles.

##### Parameters:

| Name | Type | Description |
| --- | --- | --- |
| `fontName` | String | Font name or family. Example: "times" |
| `fontStyle` | String | Font style or variant. Example: "italic" |

##### Returns:

Type
:   jsPDF

#### setFontSize(size) → {jsPDF}

Source:
:   - jspdf.js, line 1806

Sets font size for upcoming text elements.

##### Parameters:

| Name | Type | Description |
| --- | --- | --- |
| `size` | Number | Font size in points. |

##### Returns:

Type
:   jsPDF

#### setFontStyle(style) → {jsPDF}

Source:
:   - jspdf.js, line 1837

Switches font style or variant for upcoming text elements,
while keeping the font face or family same.
See output of jsPDF.getFontList() for possible font names, styles.

##### Parameters:

| Name | Type | Description |
| --- | --- | --- |
| `style` | String | Font style or variant. Example: "italic" |

##### Returns:

Type
:   jsPDF

#### setLineCap(style) → {jsPDF}

Source:
:   - jspdf.js, line 2107

Sets the line cap styles
See {jsPDF.CapJoinStyles} for variants

##### Parameters:

| Name | Type | Description |
| --- | --- | --- |
| `style` | String \| Number | A string or number identifying the type of line cap |

##### Returns:

Type
:   jsPDF

#### setLineJoin(style) → {jsPDF}

Source:
:   - jspdf.js, line 2130

Sets the line join styles
See {jsPDF.CapJoinStyles} for variants

##### Parameters:

| Name | Type | Description |
| --- | --- | --- |
| `style` | String \| Number | A string or number identifying the type of line join |

##### Returns:

Type
:   jsPDF

#### setLineWidth(width) → {jsPDF}

Source:
:   - jspdf.js, line 1898

Sets line width for upcoming lines.

##### Parameters:

| Name | Type | Description |
| --- | --- | --- |
| `width` | Number | Line width (in units declared at inception of PDF document) |

##### Returns:

Type
:   jsPDF

#### setPage(page) → {jsPDF}

Source:
:   - jspdf.js, line 1170

Adds (and transfers the focus to) new page to the PDF document.

##### Example

```
doc = jsPDF()
doc.addPage()
doc.addPage()
doc.text('I am on page 3', 10, 10)
doc.setPage(1)
doc.text('I am on page 1', 10, 10)
```

##### Parameters:

| Name | Type | Description |
| --- | --- | --- |
| `page` | Number | Switch the active page to the page number specified |

##### Returns:

Type
:   jsPDF

#### setProperties(A) → {jsPDF}

Source:
:   - jspdf.js, line 1786

Adds a properties to the PDF document

##### Parameters:

| Name | Type | Description |
| --- | --- | --- |
| `A` | Object | property\_name-to-property\_value object structure. |

##### Returns:

Type
:   jsPDF

#### setTextColor(r, g, b) → {jsPDF}

Source:
:   - jspdf.js, line 2052

Sets the text color for upcoming elements.
If only one, first argument is given,
treats the value as gray-scale color value.

##### Parameters:

| Name | Type | Description |
| --- | --- | --- |
| `r` | Number | Red channel color value in range 0-255 or {String} r color value in hexadecimal, example: '#FFFFFF' |
| `g` | Number | Green channel color value in range 0-255 |
| `b` | Number | Blue channel color value in range 0-255 |

##### Returns:

Type
:   jsPDF

#### text(text, x, y, flags) → {jsPDF}

Source:
:   - jspdf.js, line 1266

Adds text to page. Supports adding multiline text when 'text' argument is an Array of Strings.

##### Parameters:

| Name | Type | Description |
| --- | --- | --- |
| `text` | String \| Array | String or array of strings to be added to the page. Each line is shifted one line down per font, spacing settings declared before this call. |
| `x` | Number | Coordinate (in units declared at inception of PDF document) against left edge of the page |
| `y` | Number | Coordinate (in units declared at inception of PDF document) against upper edge of the page |
| `flags` | Object | Collection of settings signalling how the text must be encoded. Defaults are sane. If you think you want to pass some flags, you likely can read the source. |

##### Returns:

Type
:   jsPDF

#### triangle(x1, y1, x2, y2, x3, y3, style) → {jsPDF}

Source:
:   - jspdf.js, line 1641

Adds a triangle to PDF

##### Parameters:

| Name | Type | Description |
| --- | --- | --- |
| `x1` | Number | Coordinate (in units declared at inception of PDF document) against left edge of the page |
| `y1` | Number | Coordinate (in units declared at inception of PDF document) against upper edge of the page |
| `x2` | Number | Coordinate (in units declared at inception of PDF document) against left edge of the page |
| `y2` | Number | Coordinate (in units declared at inception of PDF document) against upper edge of the page |
| `x3` | Number | Coordinate (in units declared at inception of PDF document) against left edge of the page |
| `y3` | Number | Coordinate (in units declared at inception of PDF document) against upper edge of the page |
| `style` | String | A string specifying the painting style or null. Valid styles include: 'S' [default] - stroke, 'F' - fill, and 'DF' (or 'FD') - fill then stroke. A null value postpones setting the style so that a shape may be composed using multiple method calls. The last drawing method call used to define the shape should not have a null style argument. |

##### Returns:

Type
:   jsPDF

  

Documentation generated by JSDoc 3.4.2 on Sun Oct 09 2016 11:08:27 GMT+0100 (BST) using the docdash theme.
